# Exosomes facilitate intercellular communication between uterine perivascular adipose tissue and vascular smooth muscle cells in pregnant rats

**DOI:** 10.1152/ajpheart.00322.2022

**Published:** 2022-07-29

**Authors:** Oluwatobiloba Osikoya, Spencer C. Cushen, Jennifer J. Gardner, Megan M. Raetz, Bhavani Nagarajan, Sangram Raut, Styliani Goulopoulou

**Affiliations:** ^1^Department of Physiology and Anatomy, University of North Texas Health Science Center, Fort Worth, Texas; ^2^Texas College of Osteopathic Medicine, University of North Texas Health Science Center, Fort Worth, Texas; ^3^Department of Basic Sciences, Lawrence D. Longo MD Center for Perinatal Biology, Loma Linda University School of Medicine, Loma Linda, California

**Keywords:** exosomes, perivascular adipose tissue, pregnancy, uterine arteries, vascular reactivity

## Abstract

Perivascular adipose tissue (PVAT) is distinct from other adipose depots, as it has differential gene and protein profiles and vasoactive functions. We have shown that pregnancy affects the morphology of PVAT surrounding the uterine arteries (utPVAT) differentially than the morphology of nonperivascular reproductive adipose depots (i.e., periovarian adipose tissue, OVAT). Here, we hypothesized that pregnancy modifies the profile (size and molecular mass) of exosome-like extracellular vesicles released by utPVAT (Exo-utPVAT) compared with exosome-like extracellular vesicles released by OVAT (Exo-OVAT) and that primary uterine vascular smooth muscle cells (utVSMCs) can internalize Exo-utPVAT. Our findings indicate that utPVAT from pregnant and nonpregnant rats secrete exosome-like vesicles. Exo-utPVAT from pregnant rats were smaller (i.e., molecular size) and heavier (i.e., molecular mass) than those from nonpregnant rats, whereas pregnancy did not affect the size of Exo-OVAT. Immunocytochemistry and confocal microscopy showed that primary utVSMCs internalized Exo-utPVAT (both tissues from the same pregnant rat) labeled by the lipophilic tracer DiO. Treatment of isolated uterine arteries with Exo-utPVAT did not affect relaxation responses to acetylcholine in pregnant or nonpregnant rats. Collectively, these findings demonstrate a novel type of intercellular communication between Exo-utPVAT and utVSMCs and indicate pregnancy modulates the morphology and cargo of Exo-utPVAT.

**NEW & NOTEWORTHY** Uterine perivascular adipose tissue secretes exosome-like vesicles, which are internalized by their adjacent uterine vascular smooth muscle cells. Consideration of the exosomal communication between adipose tissue and vascular smooth muscle cells in the uterine circulation in mathematical models and experimental designs may help us to improve understanding of mechanisms underlying uterine artery adaptive responses to a healthy pregnancy and during pregnancy complications.

## Graphical abstract

**Figure:**
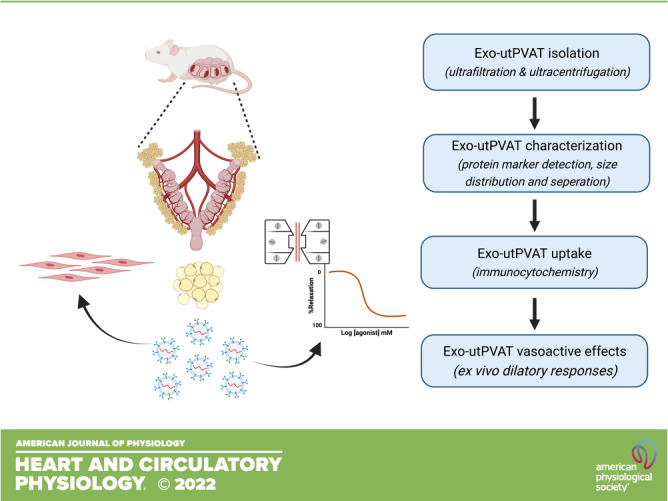

## INTRODUCTION

Perivascular adipose tissue (PVAT) contributes to vascular tone regulation via its ability to release products with paracrine vasoactive properties (1–4). We recently showed that perivascular adipose tissue surrounding uterine arteries (utPVAT) contributed to increases in uterine artery blood flow in pregnant but not in nonpregnant rats (5). In addition, utPVAT-conditioned media reduced relaxation responses to acetylcholine (ACh) in isolated uterine arteries (5, 6). These effects were explained in part by pregnancy-induced changes in responsiveness of the uterine vascular wall to utPVAT-derived factors (5).

Exosomes have a critical role in the paracrine actions of nonperivascular adipose tissues because they carry cargo that modifies epigenetic and signaling pathways in target cells (7–9). Recent studies reported that similar to nonperivascular adipose tissues (10–12), PVAT also secretes extracellular vesicles (13–15). Our previous studies have shown that pregnancy, which is a physiological state of dynamic changes and adaptations, affects PVAT morphology, genotypic profile, and vasodilatory properties (5, 6). Interestingly, pregnancy differentially affected utPVAT morphology compared with periovarian adipose tissue (OVAT), which is a nonperivascular adipose tissue surrounding reproductive organs and thus in proximity with utPVAT.

Based on previous findings, we sought to investigate whether utPVAT secretes exosome-like extracellular vesicles, if pregnancy modulates the morphology of these vesicles and if such vesicles are internalized by adjacent uterine vascular smooth muscle cells (utVSMCs). Furthermore, we sought to determine if exosome-like extracellular vesicles released by utPVAT (Exo-utPVAT) could explain in part the effects of utPVAT-derived factors on uterine artery dilation that we had seen in our previous work and if the effects of pregnancy on Exo-utPVAT differ from those on vesicles released from nonperivascular adipose depots. We selected OVAT as a reference tissue to address the latter because of its localization (reproductive adipose depot). We hypothesized that pregnancy modifies the profile of exosome-like extracellular vesicles released by utPVAT compared with OVAT, and utVSMCs internalize exosomes released from its adjacent utPVAT.

## MATERIALS AND METHODS

### Animals and Experimental Design

Every protocol was conducted in agreement with the *Guide for the Care and Use of Laboratory Animals* of the National Institutes of Health and the ARRIVE guideline (16). These protocols were approved by the Institutional Animal Care and Use Committee of the University of North Texas Health Science Center (IACUC No. 2017-0042).

Male and virgin female Sprague-Dawley rats were purchased from ENVIGO (Indianapolis, IN). Upon arrival, the animals were acclimatized for a week before experiments began and housed in standard conditions as previously described (5). Female rats were double housed, whereas male rats were single housed. All animals were fed with standard chow and tap water ad libitum. Females were placed with males overnight for mating. *Gestational day 1* (*GD1*; term = 22–23 days) was defined as the day we identified spermatozoa in vaginal smears (5, 17). Nonpregnant rats were tested at diestrus.

Final experiments were performed in pregnant (*n* = 24) and nonpregnant rats (*n* = 16) between the ages of 18–24-wk old. Male rats (15–26 wk of age) were used only for mating. Euthanasia and tissue harvest of all animals were performed on *GD16* (5, 6) between 8:00 AM and 10:00 AM. PVAT tissue cultures were immediately established after tissue harvest, and extracellular vesicles were isolated the next day.

### Tissue Harvest

**Figure 1.**
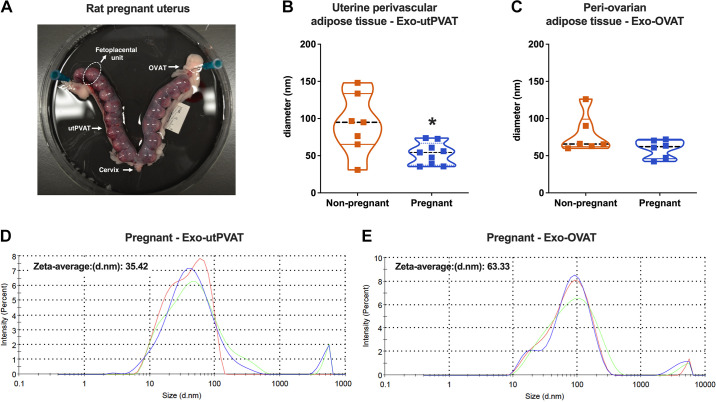
Size distribution of exosome-like extracellular vesicles in reproductive perivascular and nonperivascular adipose tissue from nonpregnant and pregnant rats. *A*: adipose tissue surrounding uterine arteries (perivascular adipose tissue, utPVAT) and ovaries (periovarian adipose tissue, OVAT) in a gravid rat (gestational age, 16 days, term = 22–23 days). Size distribution of exosome-like extracellular vesicles derived from utPVAT (Exo-utPVAT; nonpregnant, *n* = 7; pregnant, *n* = 9; *B*) and OVAT (Exo-OVAT; nonpregnant, *n* = 6; pregnant, *n* = 6; *C*) of nonpregnant and pregnant rats. Representative graphical displays of intensity particle size distribution (PSD) with logarithmic *x*-axis and linear *y*-axis from measurements of vesicles derived from utPVAT (Exo-utPVAT; *D*) and OVAT (Exo-OVAT; *E*) from pregnant rats. Values are presented as medians [interquartile ranges, IQR]. *A*: Student’s *t* test with Welch’s correction, *P = 0.04. *B*: Mann–Whitney test, *P* = 0.31. Exo-OVAT, exosome-like extracellular vesicles derived from periovarian adipose tissue.

Pregnant rats (*GD16*) and nonpregnant rats were euthanized using isoflurane overdose followed by cutting the diaphragm and removing their hearts. Uterine artery remodeling in rodents occurs between *gestational days 15–18* (18, 19). Furthermore, our previous studies on the vasoactive effects of PVAT and the effects of pregnancy on PVAT protein/gene profile have been performed on *GD16* (5, 6). Thus, in the present study, all pregnant rats were tested on *GD16*. Fetuses were euthanized via decapitation. Upon removal of the surrounding mesometrium, ovarian adipose tissue (OVAT), and utPVAT (Fig. 1*A*), segmented uterine arteries were isolated and used immediately for ex vivo vascular reactivity experiments or processed for isolation of utVSMCs.

### Isolation of Exosome-Like Extracellular Vesicles from OVAT and utPVAT Tissue Culture Media

We isolated and purified exosome-like extracellular vesicles from OVAT and utPVAT tissue culture media using ultrafiltration and ultracentrifugation (20). After OVAT and utPVAT were weighed, tissues were minced in a tissue culture hood and placed in six-well plates (Cat. No. 3516, Corning, NY) with 5 mL sterile exosome-free Dulbecco’s modified Eagles medium (DMEM, Cat. No. 10567-014, Gibco, Thermo Fisher Scientific, Waltham, MA) supplemented with 100 U/mL penicillin and 100 µg/mL streptomycin (Pen-Strep; Cat. No. 15140-122, Gibco, Thermo Fisher Scientific) and 10% exosome-free fetal bovine serum (FBS; Cat. No. 26140095, Gibco, Thermo Fisher Scientific). DMEM and FBS were depleted of exosomes via ultracentrifugation. Minced adipose tissues were then cultured at 37°C in a humidified incubator (HeraCell 150i, Model No. 1335, Thermo Fisher Scientific) containing 5% CO_2_ for 24 h. At 24 h, tissue culture media was collected and filtered through a 0.2 µm-sized 50-mL tube top vacuum filter system (Cat. No. 430320, Corning, NY) at room temperature to remove any contaminating microvesicles and cell debris (15). Filtrates were then centrifuged in a fixed angle rotor at 100,000 *g* for at least 2 h at 4°C (Sorvall WX^+^ Ultra Series with FiberLite; F50L-8x39 rotor, Thermo Fisher Scientific) to pellet exosomes. The supernatant (Exo-free supernatant) was removed and later used as a negative control for Western blot experiments. The exosome-like vesicle pellets were resuspended in 110 µL of 0.2 µm-filtered 1× phosphate-buffered saline (PBS; 10×; Cat. No. BP399, Thermo Fisher Scientific, Waltham, MA). Samples were stored at 4°C until needed for further experimentation. Exosome-like extracellular vesicles from OVAT or utPVAT were designated as exosome-like extracellular vesicles released by OVAT (Exo-OVAT) and Exo-utPVAT, respectively.

### Characterization of Exo-utPVAT and Exo-OVAT

Size of adipose tissue-derived extracellular vesicles was detected using a particle size analyzer (Zetasizer Nano ZS system, Malvern Panalytical, Malvern, UK) according to the manufacturer’s instructions. Data were captured and analyzed with Zetasizer Nano software (Malvern Panalytical).

In addition to vesicle size, Western blot analysis was used to confirm the characterization of exosome-like extracellular vesicles by measuring content of known extracellular vesicle-associated protein markers [tumor susceptibility gene 101, TGS101, protein related to exosome biogenesis; antiprogrammed cell death 6‐interacting protein, Alix, protein associated with macrovesicle biogenesis (21)]. Exosome-free supernatants were used as negative controls. Purified exosomal samples were reconstituted in ice-cold T-PER Tissue Protein Extraction reagent, and the concentration of extracted proteins was determined with a modified bicinchoninic acid protein assay as in other studies (6, 17, 22). Proteins (30 µg) were resolved by electrophoresis on precast gradient 4%–20% SDS-PAGE gels (Cat. No. 4561094, Bio-Rad, Hercules, CA). Membranes with Exo-utPVAT (or Exo-free supernatant) were probed for the expression of Alix [Cat. No. ab186429, description: rabbit monoclonal (recombinant), dilution: 1:1,000 (23)] and TSG101 [Cat. No. ab125011, description: rabbit monoclonal (recombinant), dilution: 1:1,000 (23)]. The immunostaining was detected using horseradish peroxidase-conjugated anti-rabbit immunoglobulin G (IgG; GE Healthcare; dilution: 1:10,000) or anti-mouse IgG (GE Healthcare; dilution: 1:10,000) for 1 h at room temperature. Immunoreactive bands were visualized with an enhanced chemiluminescence detection system (ChemiDoc Imaging System, Bio-Rad) and quantified using Image Lab software (Bio-Rad).

Molecular masses of Exo-utPVAT and Exo-OVAT were determined using AKTA purifier (GE Healthcare) fast protein liquid chromatography (FPLC) system by monitoring the 280-nm wavelength. Purified Exo-utPVAT and Exo-OVAT were suspended in 1× PBS and then injected and passed through a Superose 6 (increase 10/300) column (fractionation range of 5,000–5,000,000 Da molecular mass), and 1-mL fractions were collected using an autofractionator. Known molecular mass standards (Cat. No. 28403842, GE Healthcare, Chicago, IL) were run on the FPLC systems to estimate the molecular mass of exosomes based on elution profile. Two independent samples per group were analyzed for each tissue. To ensure that the FPLC outcomes were specific to exosomes, we used as controls exosome-free supernatants generated during the exosome isolation process (ultracentrifugation). Two controls (*control 1* and *control 2*) were used and represent two independent biological samples.

### Isolation of Uterine Artery Vascular Smooth Muscle Cells

UtVSMCs from pregnant rats (*GD16*) were isolated from the main uterine arteries using an enzymatic digestion method (24). Cell pellets were resuspended in DMEM (with 10% FBS and 100 U/mL penicillin, 100 µg/mL streptomycin), and cell solutions were distributed in culture dishes and kept in a humidified incubator at 37°C (5% CO_2_). Cell culture media was replaced every 48 h. Purity of utVSMCs was tested by immunocytochemistry for positive staining of VSMC marker smooth muscle actin (anti-α-smooth muscle actin, 1:400, Sigma Cat. No. A2547; secondary: anti-mouse Alexa 488, 1:500, Abcam, Cat. No. ab150113). Anti platelet endothelial cell adhesion molecule 1 (PECAM 1; CD31, 1:3,200, Cell Signaling, Cat. No. 3528S) was used as a negative control. Cultured utVSMCs were used at *passage 2* to avoid genotypic and phenotypic drifts.

### Coculture of Exo-utPVAT and utVSMCs

utVSMCs were plated into 24-well plates (Cat. No. 3526, Corning) containing cover glasses (Cat. No. 12-545-80, Thermo Fisher Scientific) at a seeding density of 40,000 viable cells/mL (20,000 cells/well, 0.5 mL growth media/well). utVSMCs were allowed to incubate overnight for adherence and were then treated for 3 h (37°C, 5% CO_2_, and 100% relative humidity) with Exo-utPVAT (170 µg/well) stained with lipophilic tracer DiO cell-labeling solution (Invitrogen, V22886) as described in Horibe et al. (25) or Exo-free supernatant (170 µg/well) stained with DiO. Exo-utPVAT and Exo-free supernatant concentrations were determined as the concentration of protein present in the samples, measured with NanoDrop (Thermo Scientific NanoDrop 1000, Thermo Fisher Scientific) and analyzed with its corresponding NanoDrop software v 3.8.1. Exo-utPVAT and Exo-free supernatant were incubated with 5 µL of 1 mM DiO for 20 min at 37°C. Stained solutions were separated from the remaining DiO by filtering through an exosome spin column (MW 3,000, Cat. No. 4484449, Invitrogen) at 750 *g* for 2 min at room temperature (25). Imaging controls were as follows: *1*) untreated utVSMCs to determine autofluorescence of isolated cells, *2*) 170 µg/well of unlabeled Exo-utPVAT to determine autofluorescence, and *3*) DiO dye alone (5 µL of 1 mM DiO into 55 µL PBS) to ensure effective functioning of spin column removal of excess DiO. After treatment, utVSMCs were washed with PBS, fixed with 4% paraformaldehyde (PFA) for 10 min, permeabilized with 0.1% Triton X-100 for 5 min, stained with (1:1,000) Alexa Fluor 555 phalloidin (Cat. No. A34055, Invitrogen) for 30 min, and mounted with ProLong Diamond Antifade Mountant with DAPI (Cat. No. P36962, Thermo Fisher Scientific) and allowed to cure for 24 h at 4°C as described in Horibe et al. (25). All reagents and procedures were in room temperature until slides were placed at 4°C. Cells were washed with PBS (37°C) between steps. For these studies, uterine Exo-utPVAT and utVSMCs were isolated from the same timed-pregnant rat for each experiment. Two independent experiments (2 biological replicates) were performed.

Internalization of Exo-utPVAT into utVSMCs was visualized using a confocal microscope (LSM 880, Zeiss, Germany). Images were captured at ×20. Laser energy required to image DiO-labeled Exo-utPVAT-treated utVSMCs was the energy used for acquiring images for the rest of the samples from the same animal on the same experimental day. Confocal images were processed using ZEN imaging software (Zeiss, Germany). To confirm the uptake of exosomes into utVSMCs, a super-resolution *Z*-stack confocal image was generated from DiO-labeled utVSMCs treated with Exo-utPVAT.

### Vascular Reactivity Experiments

Isolated 2-mm uterine artery rings were treated with 200 µg/mL of Exo-utPVAT (26) or corresponding volume of 1× PBS (vehicle) for 3 h and incubated at 37°C, 5% CO_2_, and 100% relative humidity. After 3 h, uterine arteries were mounted onto wire myograph chambers (Danish Myo technology, Aarhus, Denmark), baseline tension was determined for individual segments, and vascular smooth muscle and endothelial integrity were assessed as in Ref. 5 and Ref. 17. To determine whether Exo-utPVAT contributes to the effects of utPVAT on ACh-induced relaxation in uterine arteries as in Osikoya et al. (5, 6), we performed concentration-response curves to ACh (10^−9^–3 × 10^−5^ M) in uterine arteries contracted with phenylephrine (1 μM). For these experiments, Exo-utPVAT and uterine arteries were from different animals, because uterine artery reactivity requires immediate use of the arteries, whereas isolation of Exo-utPVAT requires at least 24 h. Uterine arteries from pregnant rats (*GD16*) were incubated with Exo-utPVAT from gestational age-matched pregnant rats, and uterine arteries from nonpregnant rats at diestrus were incubated with Exo-utPVAT from age-matched nonpregnant rats at diestrus.

### Data Analysis

The number of animals necessary to complete the characterization of PVAT-derived exosomes and reactivity experiments was estimated using power analysis based on preliminary data and previous studies (5, 6, 27). These numbers were selected with a goal to achieve a power of 0.80–0.85 with a probability of a type I error of 0.05. Concentration-response curves were fit and analyzed using robust nonlinear regression analysis as we previously described (5). To quantify vascular responses to ACh, we calculated EC_50_ (expressed as *p*EC50: negative logarithm of EC_50_) for each sigmoidal curve, and group means were compared using an unpaired *t* test. Unpaired *t* test with Welch’s correction and Mann–Whitney test were used to compare the size of Exo-utPVAT and Exo-OVAT, respectively, between pregnant and nonpregnant rats. All statistical analyses were conducted with GraphPrism software (v. 9.4, San Diego, CA). Exact *P* values are reported.

## RESULTS

Particle size determination revealed that Exo-utPVAT had a median diameter ranging between 47 and 99 nm (Fig. 1, *B*and *C*), in line with the size range previously reported for exosomes (28). Exo-utPVAT from pregnant rats were smaller compared with Exo-utPVAT from nonpregnant rats (Fig. 1*B*), but this difference was not observed in Exo-OVAT (Fig. 1*C*). Figure 1, *D*and *E*, shows representative graphical displays of intensity particle size distribution (PSD) with logarithmic *x*-axis and the linear *y*-axis from measurements of the size of Exo-utPVAT (Fig. 1*D*) and Exo-OVAT (Fig. 1*E*) derived from pregnant rats.

**Figure 2.**
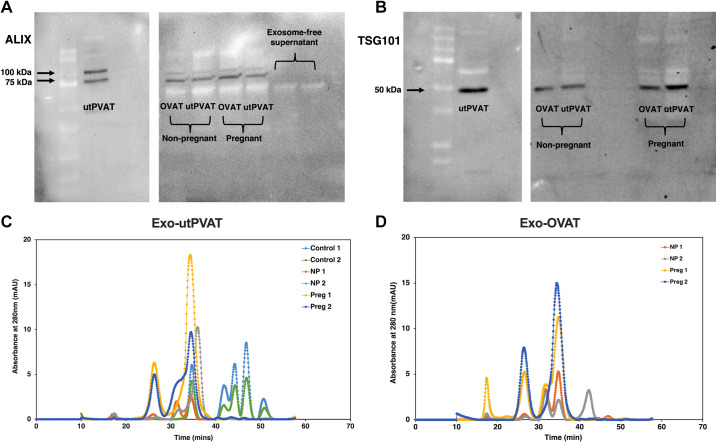
Characterization of exosome-related protein markers and molecular masses in vesicles isolated from adipose-tissue culture media. Protein content of Alix (100 and 75 kDa; *A*) and TSG101 (∼50 kDa; *B*) in samples of Exo-utPVAT and Exo-OVAT derived from nonpregnant and pregnant rats. Representative chromatograms fast protein liquid chromatography (FPLC) for Exo-utPVAT (*C*) and Exo-OVAT samples (*D*), demonstrating time at which the protein eludes in the FPLC column. Exo-OVAT, exosome-like extracellular vesicles derived from periovarian adipose tissue; Exo-utPVAT, exosome-like extracellular vesicles derived from uterine perivascular adipose tissue; utPVAT, uterine perivascular adipose tissue.

Vesicles isolated and purified from OVAT and utPVAT expressed exosome-associated proteins TSG101 and Alix (Fig. 2, *A*and *B*). With FPLC, two major protein peaks were recognized in chromatograms from all exosome samples (Fig. 2, *C*and *D*). Control samples (Fig. 2*C*) did not show these peaks and most of the peaks for the control samples were concentrated at late elution times beyond 40 min suggesting small molecular mass proteins/complexes. The two major peaks noted at exosome samples were with elution times of 26.3 and 34.4 min in the chromatogram. These elution times were used to estimate the molecular mass of exosomes based on the known molecular mass of standard proteins that were ran on the FPLC system. Based on this extrapolation, molecular mass of Exo-utPVAT from pregnant rats was ∼95–100 and ∼2,000 kDa, whereas molecular mass of Exo-utPVAT samples from nonpregnant rats was ∼97, ∼250–310, and ∼2,000 kDa. The molecular masses of Exo-OVAT were ∼80–100 and ∼2,000 kDa in samples from pregnant rats and ∼80 kDa, ∼270 kDa, and ∼2,000 kDa in samples from nonpregnant rats. In Exo-utPVAT samples, the protein peak corresponding to ∼2,000 kDa was ∼9.2× greater in samples from pregnant compared with nonpregnant rats, whereas in Exo-OVAT, the same peak was 13.8× greater in samples from pregnant compared with nonpregnant rats.

**Figure 3.**
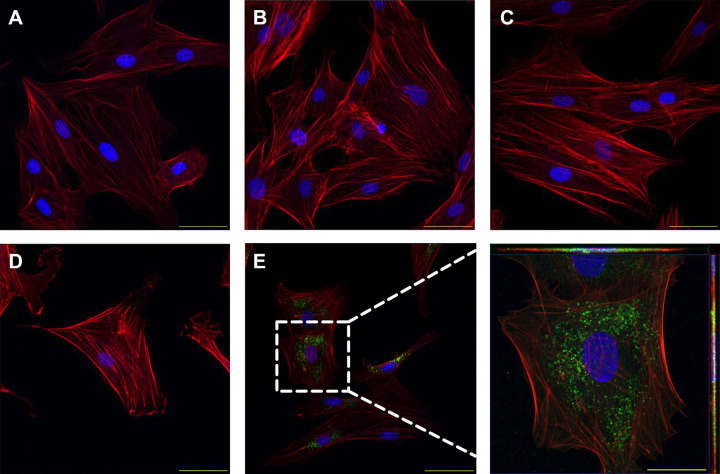
Trafficking of uterine perivascular adipose tissue-derived exosome-like vesicles into adjacent uterine vascular smooth muscle cells in a pregnant rat. Immunocytochemical images of untreated utVSMCs (*A*), utVSMCs treated with unlabeled exosomes (*B*), utVSMCs treated with membrane staining solution DiO (*C*), utVSMCs treated with exosome-free supernatant (*D*), and utVSMCs treated with labeled Exo-utPVAT (*E*). *Inset*: Exo-utPVAT are labeled green (63× *Z*-stack max intensity). Scale bar = 200 µm. Tissues were derived from a pregnant rat (gestational age = 16 days, term = 22–23 days). Exo-utPVAT, exosome-like extracellular vesicles derived from uterine perivascular adipose tissue; utPVAT, uterine perivascular adipose tissue; utVSMCs, uterine vascular smooth muscle cells.

Internalization of utPVAT-derived exosome-like extracellular vesicles by primary utVSMCs was investigated using immunocytochemistry and confocal microscopy. Control experiments with untreated utVSMCs (Fig. 3*A*), utVSMCs treated with unlabeled Exo-utPVAT (Fig. 3*B*), and utVSMCs treated with exosome-free supernatant (Fig. 3*C*) showed no green fluorescence intensity, which was observed in utVSMCs cocultured with DiO-labeled Exo-utPVAT from the same pregnant rat (Fig. 3*E*).

**Figure 4.**
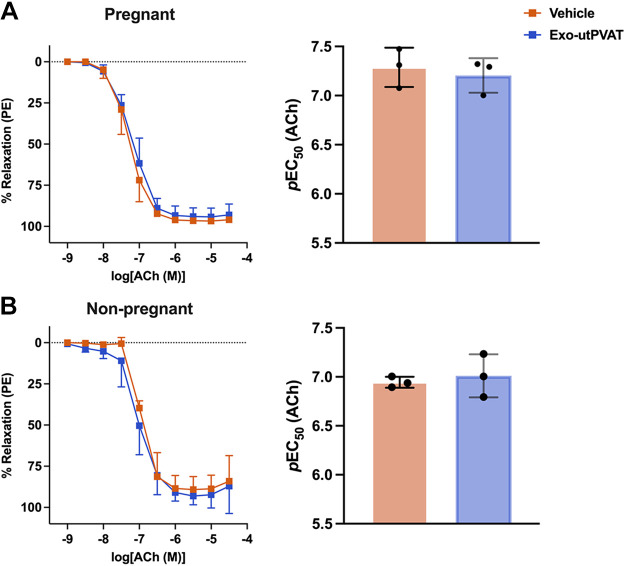
Uterine perivascular adipose tissue-derived exosome-like vesicles (Exo-utPVAT) does not affect relaxation responses to acetylcholine (Ach). Concentration-response curves to ACh and corresponding *p*EC50 in main uterine arteries from pregnant (*A*) and nonpregnant (*B*) rats. Student’s *t* test: *P* = 0.64 (*A*) and *P* = 0.62 (*B*); *n* = 3/group. Values are means ± SD.

Incubation of uterine arteries with Exo-utPVAT did not affect ACh-induced relaxation in pregnant (*p*EC50, Veh, 7.28 ± 0.20 vs. Exo-utPVAT, 7.21 ± 0.18, *P* = 0.62) or in nonpregnant rats (*p*EC50, Veh, 6.45 ± 0.06 vs. Exo-utPVAT, 7.01 ± 0.22, *P* = 0.64; Fig. 4). Contractile responses to a bolus of phenylephrine (1 µM) was not affected by exposure to Exo-utPVAT in pregnant (Veh, 15.56 ± 8.69 mN vs. Exo-utPVAT, 17.60 ± 4.96 mN, *P* = 0.74) or in nonpregnant rats (Veh, 16.39 ± 1.43 mN vs. Exo-utPVAT, 16.07 ± 0.99 mN, *P* = 0.76).

## DISCUSSION

Perivascular adipose tissue is a metabolically active adipose depot that secretes adipocytokines with paracrine actions on the adjacent vascular wall (5, 29–31). A recent study by Li et al. (15) demonstrated that mouse mesenteric PVAT secretes extracellular vesicles that carry their cargo into VSMCs. This investigation added extracellular vesicles in the list of utPVAT-derived products and opened new horizons in the field of PVAT biology (15). The present study provides new knowledge about intercellular communication between PVAT and maternal vessels in the uterine circulation, which may be relevant for the establishment of blood flow to the growing fetus during pregnancy.

Previous studies have shown increases in maternal nonperivascular adipose tissue mass (27, 32), adipocyte cell number and size (27, 33), fat accretion, and lipid metabolism (34) in response to pregnancy in humans and in rodent experimental models. With regard to PVAT, we reported that pregnancy differentially modified the cellularity of utPVAT and nonperivascular adipose tissue (i.e., OVAT) such that the adipocytes of OVAT were larger in pregnant compared with nonpregnant rats on *gestational day 16* (third trimester of rat pregnancy; 5), whereas there were no differences in the size of utPVAT adipocytes between groups (5). Furthermore, we found that pregnancy downregulated gene expression of metabolic adipokines in utPVAT (5) but not in aortic PVAT (5) or OVAT (unpublished observations). These results suggest that utPVAT has a different phenotypic and gene expression response to pregnancy compared with nonperivascular adipose depots.

The differential effects of pregnancy on types of adipose tissue depots were further demonstrated in the present study because pregnancy reduced the size of exosomes from utPVAT but had no effect on the size of exosomes from OVAT. Size separation of purified exosomes from utPVAT and OVAT revealed that the relative abundance of vesicles with molecular mass of ∼2,000 kDa was greater in the samples from pregnant animals. In addition, Exo-utPVAT and Exo-OVAT samples from pregnant rats did not have vesicles of ∼250–310 kDa. These data indicate that pregnancy induces differential effects on phenotypic and gene expression of maternal utPVAT and non-PVAT tissues and also modulates the morphology and possibly the content of utPVAT-derived exosome-like extracellular vesicles. To the best of our knowledge, this is the first report of a physiological condition that reduces the size of exosomes but increases their molecular mass.

When we investigated the communication between uterine Exo-utPVAT and utVSMCs, we discovered that primary utVSMCs internalized Exo-utPVAT. In our previous work, we reported that utPVAT-derived factors suppressed uterine artery relaxation responses to ACh and this effect was specific to pregnancy (5). Here, we sought to investigate whether these effects were attributed to Exo-utPVAT but found that the presence of Exo-utPVAT did not have an effect on uterine artery relaxation. Since we have no evidence to demonstrate uptake of Exo-utPVAT from the isolated uterine arteries, we should consider the possibility that Exo-utPVAT was degraded in the myograph chamber during the incubation period. However, our cell culture experiments suggest that utVSMCs have internalized exosomes after 3 h of coculture with them. This time period is the same as the one in the vascular reactivity studies. Others have shown internalization of Exo-utPVAT by primary VSMCs even after 18 h of coculture (15). Our findings are interpreted with caution in the context of pregnancy because although primary utVSMCs were used near isolation (*passage 2*), potential genotypic or phenotypic drifts should not be disregarded.

During pregnancy, the uterine vascular bed becomes a low-resistance shunt that allows increases in uterine blood flow to the growing fetus, the newly developed placenta, and the myometrium (35). These changes are brought by an increase in vascular dilatory responses and extensive regional remodeling (36). Li et al. (15) demonstrated that extracellular vesicles from mesenteric adipose tissue and isolated mesenteric adipocytes carried miRNAs that affected VSMC proliferation and migration in vitro. Thus, the involvement of utPVAT-derived exosome-like vesicles in uterine vascular remodeling during pregnancy is an exciting possibility.

In conclusion, utPVAT secretes exosome-like vesicles, which are internalized by adjacent utVSMCs. To the best of our knowledge, this is the first study to demonstrate exosomal interaction between tunica media and tunica adiposa in uterine arteries. Our findings are timely and highly relevant to recent advancements in the field of uterine vascular adaptations to pregnancy. Allerkamp et al. (37) used mathematical modeling to demonstrate distinct functional and structural profiles of uterine arteries at the pregnant compared with the nonpregnant states. Our findings suggest that the interaction between utPVAT and utVSMCs via exosomes should be considered in pregnancy-specific multiscale models that aim to improve understanding of mechanisms underlying uterine artery adaptive responses to pregnancy.

## GRANTS

This research was supported in part by National Institutes of Health Grants R01HL0146562 (to S.G.) and T32AG020494 (to S.C.C.) and a Basic Research Seed Funding Program from the University of North Texas Health Science Center (to S.G.).

## DISCLOSURES

No conflicts of interest, financial or otherwise, are declared by the authors.

## AUTHOR CONTRIBUTIONS

O.O., S.C.C., S.R., and S.G. conceived and designed research; O.O., S.C.C., J.J.G., M.M.R., B.N., and S.R. performed experiments; O.O., B.N., and S.G. analyzed data; O.O., S.C.C., J.J.G., M.M.R., S.R., and S.G. interpreted results of experiments; O.O., S.C.C., B.N., S.R., and S.G. prepared figures; O.O. and S.G. drafted manuscript; O.O., S.C.C., J.J.G., M.M.R., B.N., S.R., and S.G. edited and revised manuscript; O.O., S.C.C., J.J.G., M.M.R., B.N., S.R., and S.G. approved final version of manuscript.
